# General Medicine

**Published:** 1903-09

**Authors:** 


General Medicine.—Edited by Frank Billings, M. S., M. D. Head of the Medical Department, and Dean of the Faculty of Rush Medical College; and J. H. Salisbury, M. D., Professor of Medicine, Chicago Clinical School. Being Vol. VI of the Practical Medicine Series of Year Books of ten volumes, issued monthly. Price, per volume, $1.50; per series, $7.50. Published by the Year-Book Publishers, 40 Dearborn Street, Chicago.

This volume deals with the diseases of the alimentary tract and the summer diseases. Several new points are suggested for the diagnosis of typhoid fever and para-typhoid fever. In the former, blood cultures have been found to yield positive results in a large enough per cent of cases to make them a practical test. Photography has revealed the rose spots before they could be seen or felt. The relation of sunlight to malaria is discussed, as is also the serum treatment of cholera. Recent experiments on the healthy stomach have suggested several aids for the diagnosis of disease in that organ. Of these the stomach tube is highly recommended. The misuse of the tube, however, is warned against. New suggestions are given on diet in intestinal diseases. Not a few good ideas are brought out on the diagnosis and treatment of disease of the liver and pancreas.

The books of the series are thorough, up-to-date, seasonable, convenient in size, and low in price, and every progressive physician should own them. J. M. L.

